# Adaptive Advantage of the Emerging Seasonal Influenza A (H3N2) Subclade K: Enhanced Receptor Binding Capacity and Immune Escape Potential

**DOI:** 10.1002/mco2.70953

**Published:** 2026-08-19

**Authors:** Lulu Zhang, Tingbi Zhao, Xiaoqian Pan, Zhaomin Feng, Daitao Zhang, Quanyi Wang, Peng Yang

**Affiliations:** ^1^ Beijing Key Laboratory of Surveillance, Early Warning and Pathogen Research on Emerging Infectious Diseases, Beijing Research Center for Respiratory Infectious Diseases Beijing Center for Disease Prevention and Control Beijing China; ^2^ School of Public Health Capital Medical University Beijing China

1

Dear editor:

Emerging influenza variants continue to drive waves of flu globally. Notably, the influenza A (H3N2) subclade K (J.2.4.1) is currently the dominant strain during the 2025–2026 Northern Hemisphere influenza season, spreading rapidly across Asia, Europe, and North America [1]. These variants exhibit multiple mutations in the hemagglutinin (HA) protein, including key substitutions in regions of antigenic sites A, B, and D, as well as the receptor binding site (RBS) (Figure 1A). Notably, glycosylation changes at positions 135 (loss) and 144 (gain) may contribute to its enhanced adaptability. The rapid spread of subclade K, along with early reports of flu outbreaks in countries such as Japan, the United Kingdom (UK) and the United States (USA), has prompted public health agencies to prioritize surveillance and vaccine updates [2, 3]. However, the biological characteristics of the newly emerging subclade K remain unclear.

**FIGURE 1:**
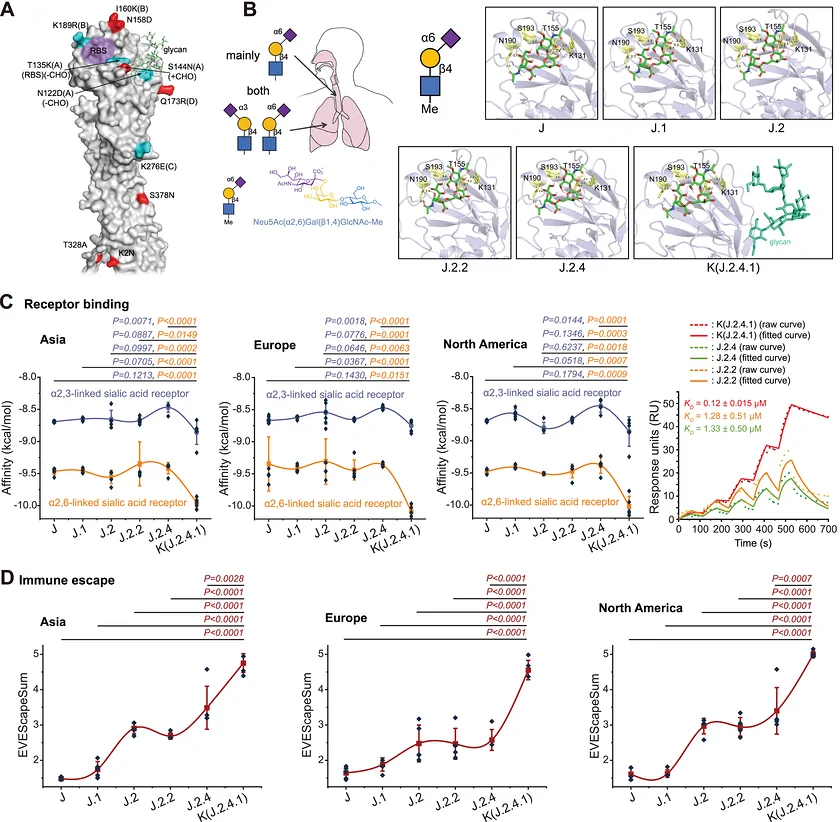
Adaptive advantage of the emerging seasonal influenza A (H3N2) subclade K. (A) The mutation of the hemagglutinin (HA) protein in influenza A (H3N2) subclade K. Red indicates the new mutation sites in subclade K compared to J.2.4. RBS, receptor binding site; ±CHO, gain or loss of glycosylation. (B) Molecular docking simulation of the binding of HA proteins from different subclades (J/J.1/J.2/J.2.2/J.2.4/K) to human α2,6‐linked sialic acid receptors. (C) Binding affinities of HAs from different influenza viruses toward α2,3‐ and α2,6‐linked sialic acid receptors. Molecular docking simulation results are presented on the left panel, surface plasmon resonance (SPR) results for α2,6‐linked sialic acid receptors are shown on the right panel. *K*_D_ values are from three independent replicates. (D) Dynamic changes in immune escape potential among different influenza virus subclades.

In this study, we retrieved all available HA sequences of influenza A (H3N2) virus belonging to clade J (2a.3a.1) from the Global Initiative on Sharing All Influenza Data (GISAID) platform. These sequences were geographically sourced from Asia, Europe, and North America, spanning the period from June 6, 2020 to January 16, 2026. A total of 62,080 HA sequences were obtained, including 11,014 sequences from the 2025–2026 influenza season. Among these, 9,795 (88.93%) were classified as subclade K. We randomly selected 5 viral sequences from each subclade (J, J.1, J.2, J.2.2, J.2.4, K) in each region of the three areas, totaling 90 viral sequences for the study of biological characteristics.

Using AlphaFold3, we identified substantial protein conformational differences between subclade K and other variants in clade J [4]. Protein structure alignment analysis revealed a progressive increase in structural divergence between subclade K and the following variants: J (average root mean square deviation (RMSD): 1.906 Å), J.1 (1.914 Å), J.2 (1.853 Å), J.2.2 (2.167 Å), and J.2.4 (2.482 Å). Notably, the RMSD between subclade K and the 2025–2026 Northern Hemisphere influenza vaccine reference strain (A/Croatia/10136RV/2023 (H3N2)‐like virus) reached 2.573 Å. These findings suggest that subclade K has undergone significant structural alterations, potentially leading to antigenic mismatch with the current vaccine composition.

To study the receptor binding capability of subclade K, we employed molecular docking techniques to simulate the binding processes of the virus with both α2,6‐linked sialic acid receptors (the primary receptor for human influenza viruses, present in the human upper respiratory tract) and α2,3‐linked sialic acid receptors (predominantly found in the avian intestinal tract and the human lower respiratory tract, serving as the primary receptor for avian influenza viruses) (Figure 1B). The results suggested that, compared to all other subclades, the K variant displays enhanced binding affinity to α2,6‐linked sialic acid receptors on human epithelial cells (*p* < 0.0001) (Figure 1C). For α2,3‐linked sialic acid receptors, the K variant showed a statistically significant increase in affinity compared to the previous circulating strain J.2.4 (*p* < 0.05). Consistent conclusions based on sequences from Asia, Europe and North America suggest the universality of this finding (Figure 1C). We used surface plasmon resonance (SPR) assays to verify the binding affinity between influenza HA proteins and sialic acid receptors. The subclade K displayed stronger binding affinity to α2,6‐linked sialic acid receptor (*p* < 0.05) but lacks detectable binding to the α2,3‐linked sialic acid receptor (Figure 1C). The enhanced receptor binding capacity of the K variant may contribute to its epidemiological fitness in human populations.

We further employed the EVEscape deep learning model to assess viral escape. This model combined fitness predictions from a deep learning model of historical sequences with biophysical and structural information. The results indicated that significantly elevated immune escape potential for subclade K compared to other variants in clade J (the average escape score increased by 3.02, 2.72, 1.71, 1.77 and 1.51 times compared to subclade J, J.1, J.2, J.2.2, and J.2.4 respectively) (*p* < 0.0001) (Figure 1D). According to the report released by WHO, post‐infection ferret antisera raised against northern hemisphere 2025–2026 influenza season vaccine viruses showed poor recognition of viruses from subclade K [5]. This suggests that the subclade K evades pre‐existing immunity more effectively, thereby contributing to its rapid spread. These findings align with observed epidemiological trends, hinting at the value of AI‐driven tools in predicting viral evolution and informing public health strategies.

Overall, this study adopted a strategy combining computational prediction with in vitro functional assays, and suggests that the currently prevalent subclade K possesses enhanced receptor binding capacity and elevated immune escape potential, providing additional molecular evidence for the evolutionary variation and epidemic risk assessment of H3N2 viruses. The apparent adaptive advantages of subclade K point toward the need for continuous monitoring and proactive prevention and control strategies, including enhanced influenza vaccination and timely use of antiviral drugs. The limitation of this study lies in the fact that only the correlation trend between antigenic variation and immune escape was observed at the overall molecular level, while the specific key antigenic sites that determine it have not yet been mapped. In future work, we will employ approaches including site‐directed mutagenesis of epitopes, structural biological analysis, and monoclonal antibody binding assays to further precisely identify the key antigenic sites and better elucidate the molecular mechanisms underlying immune escape. In addition, NA protein sequencing and functional tests are beyond the scope of current research, and proposed paired HA–NA characterization as a future research priority.

## Author Contributions

**Lulu Zhang**: writing – original draft (lead); formal analysis (lead); methodology (lead); writing – review and editing (equal). **Tingbi Zhao**: writing – original draft (equal); resources (lead); visualization (lead); writing – review and editing (equal). **Xiaoqian Pan**: investigation (lead); writing – review and editing (equal). **Zhaomin Feng**: writing – review and editing (equal). **Daitao Zhang**: writing – review and editing (equal). **Quanyi Wang**: conceptualization (supporting); writing – review and editing (equal); **Peng Yang**: conceptualization (lead); writing – original draft (supporting); writing – review and editing (equal). All authors have read and approved the final version of the manuscript.

## Funding

This work was supported by the Prevention and Control of Emerging and Major Infectious Diseases‐National Science and Technology Major Project (grant no.: 2025ZD01901702), the Capital's Funds for Health Improvement and Research (grant no.: 2026‐1G‐3012), and the Beijing Research Center for Respiratory Infectious Diseases Project (grant no.: BJRID2026‐006).

## Ethics Approval

This study only analyzed public influenza virus sequences from GISAID, without any human clinical data or identifiable participant information. No ethical approval was needed.

## Conflicts of Interest

The authors declare no conflicts of interest.

## Supporting information




**Supplementary File 1**: mco270953‐sup‐0001‐SuppMat.docx

## Data Availability

The influenza sequence data used in this study were all sourced from the GISAID database. Detailed database identifiers were listed in the Supporting Information. The experimental data of SPR included in this study are available upon request from the corresponding author.
