# Supplementary material for: Adaptive Advantage of the Emerging Seasonal Influenza A (H3N2) Subclade K: Enhanced Receptor Binding Capacity and Immune Escape Potential
Source: MedComm (2020). 2026 Aug 19;7(9):e70953. doi: 10.1002/mco2.70953 (PMC13490912; doi:10.1002/mco2.70953)
Supplement: Supplementary file 1 [file MCO2-7-e70953-s001.docx]

Supplementary materials

Adaptive advantage of the emerging seasonal influenza A (H3N2) subclade K: enhanced receptor binding capacity and immune escape potential

Lulu Zhang^12^*, Tingbi Zhao^12^*, Xiaoqian Pan^12^, Zhaomin Feng^12^, Daitao Zhang^12^, Quanyi Wang^12†^, Peng Yang^12†^

Correspondence to: Peng Yang [(yangpengcdc@163.com)](mailto:(yangjt@pumc.edu.cn))

Quanyi Wang (wangqy@bjcdc.org)

^1^Beijing Key Laboratory of Surveillance, Early Warning and Pathogen Research on Emerging Infectious Diseases, Beijing Research Center for Respiratory Infectious Diseases, Beijing Center for Disease Prevention and Control, Beijing, China;

^2^School of Public Health, Capital Medical University, Beijing, China.

**This PDF file includes:**

Supplementary Materials and Methods

Materials and Methods

**Data source**

We collected all available HA sequences of influenza A (H3N2) virus belonging to clade J from the GISAID database during the period from June 6, 2020, to January 16, 2026. Sequences were restricted to those isolated from Asia, Europe, and North America. A random sampling strategy was then applied: 30 sequences were randomly selected from each region, covering six subclades (J, J.1, J.2, J.2.2, J.2.4, and K), with five sequences per subclade, resulting in a total of 90 sequences. These sequences were subsequently used to analyze receptor-binding properties and the potential for immune escape. The GISAID accession numbers of the 90 sequences are listed below: EPI_ISL_16937239, EPI_ISL_18891563, EPI_ISL_19820840, EPI_ISL_19820846, EPI_ISL_20246818, EPI_ISL_20244200, EPI_ISL_16937237, EPI_ISL_16937246, EPI_ISL_16937250, EPI_ISL_16937248, EPI_ISL_18891569, EPI_ISL_18891525, EPI_ISL_18891587, EPI_ISL_18891599, EPI_ISL_19820835, EPI_ISL_20078882, EPI_ISL_20078881, EPI_ISL_20078886, EPI_ISL_19820847, EPI_ISL_19820844, EPI_ISL_20078884, EPI_ISL_20078890, EPI_ISL_20246800, EPI_ISL_20246801, EPI_ISL_20226416, EPI_ISL_20258884, EPI_ISL_20317217, EPI_ISL_20317216, EPI_ISL_20317214, EPI_ISL_20317212, EPI_ISL_16744259, EPI_ISL_19168782, EPI_ISL_19759103, EPI_ISL_19759551, EPI_ISL_19628073, EPI_ISL_20267728, EPI_ISL_16384492, EPI_ISL_18939973, EPI_ISL_18939979, EPI_ISL_18939984, EPI_ISL_18607611, EPI_ISL_18607613, EPI_ISL_18607602, EPI_ISL_18607605, EPI_ISL_19759484, EPI_ISL_19759486, EPI_ISL_19759533, EPI_ISL_19759532, EPI_ISL_19759545, EPI_ISL_19759500, EPI_ISL_19628525, EPI_ISL_19628524, EPI_ISL_19852663, EPI_ISL_19852709, EPI_ISL_19852661, EPI_ISL_19852698, EPI_ISL_20315256, EPI_ISL_20315255, EPI_ISL_20315254, EPI_ISL_20315252, EPI_ISL_17414427, EPI_ISL_18506015, EPI_ISL_19818766, EPI_ISL_19818781, EPI_ISL_19907042, EPI_ISL_20250042, EPI_ISL_17414431, EPI_ISL_17414432, EPI_ISL_19057007, EPI_ISL_17244503, EPI_ISL_18506012, EPI_ISL_18506019, EPI_ISL_18506016, EPI_ISL_18506017, EPI_ISL_19818782, EPI_ISL_19818780, EPI_ISL_19818779, EPI_ISL_19818776, EPI_ISL_19818778, EPI_ISL_19818775, EPI_ISL_19818773, EPI_ISL_19818770, EPI_ISL_20054876, EPI_ISL_20190082, EPI_ISL_20237022, EPI_ISL_20250034, EPI_ISL_20296999, EPI_ISL_20293158, EPI_ISL_20293154, EPI_ISL_20293145.

**Procedures**

***Biotinylation of sialoglycans***

Biotinylated α2,3- and α2,6-linked sialoglycan were prepared via amine-reactive conjugation. The α2,3-linked sialoglycan (Neu5Acα(2-3)Galβ(1-4)Glcβ-biotin) and α2,6-linked sialoglycan (Neu5Acα(2-6)Galβ(1-4)GlcNAcβ-biotin) were individually synthesized using corresponding amino-terminated sialoglycans of Neu5Acα(2-3)Galβ(1-4)Glc-β-propylamine (TCI, Catalog no. N1258) and Neu5Acα(2-6)Galβ(1-4)GlcNAc-β-ethylamine (CAS no. 2765625-81-6, TCI, Catalog no. N0950). For the biotinylation reaction, 5 mM amino sialoglycans and 3.2 mM Sulfo-NHS-LC-biotin (CAS no. 191671-46-2) were fully dissolved in PBS. The reaction mixture was incubated with continuous shaking at room temperature for 4 h. Residual NHS esters were subsequently quenched by adding 32 mM glycine, followed by a further 30 min reaction.

The biotinylation of sialoglycans was verified by liquid chromatography-mass spectrometry (LC-MS). Meanwhile, Glycine-biotin negative control was prepared using an identical reaction procedure, where 5 mM glycine was used to replace amino sialoglycans to eliminate the interference of biotin and non-specific binding.

***Surface plasmon resonance (SPR)***

SPR measurements was performed on Biacore 8K, with PBST (137 mM NaCl, 2.7 mM KCl, 10 mM Na2HPO4, 1.8 mM KH2PO4, 0.005 % Tween 20) serving as running buffer. Biotinylated sialoglycans, including Neu5Acα(2-3)Galβ(1-4)Glcβ-biotin (5μM) and Neu5Acα(2-6)Galβ(1-4)GlcNAcβ-biotin (5μM), were immobilized at SA sensor chip. Glycine-biotin was fixed on chip as a negative control for background correction. Recombinant H3N2 HA proteins of subclade J.2.2 (Catalog no. DRA343) and J.2.4 (Catalog no. DRA344) were obtained from Novoprotein. The HA protein of subclade K (J.2.4.1), including residues Gln17 to Asp529, was expressed in 293 cells based on the sequence from strain A/Ohio/PLMI-LYTJD-00594/2025 (GISAID accession number, EPI_ISL_20267728). The proteins were serially diluted to indicated concentrations with PBST running buffer. For the association, diluted proteins were injected over flow cells 1 and 2 at a flow rate of 30 μL/min for 120 s. Subsequently, buffer flow was maintained for 180 s to facilitate molecular dissociation at a flow rate of 30 μL/min. The sensor surface was stabilized for 1200 s before the subsequent injections. The *K*_D_ value was evaluated using Biacore 8K software (version: 3.0.12.15655).

**Statistical analyses**

The student *t* test was performed to compare sialic acid receptor binding affinities and immune escape scores of HA proteins across different H3N2 subclades. Spline functions were applied to smooth the values of receptor binding affinity and immune escape score. A two-sided *P* value < 0.05 was considered statistically significant. All statistical analyses were conducted using R (version 3.6.1) and GraphPad Prism (version 9.5.0).
